# Rutile TiO_2_ Mesocrystals/Reduced Graphene Oxide with High-Rate and Long-Term Performance for Lithium-Ion Batteries

**DOI:** 10.1038/srep08498

**Published:** 2015-02-17

**Authors:** Tongbin Lan, Heyuan Qiu, Fengyan Xie, Jie Yang, Mingdeng Wei

**Affiliations:** 1 State Key Laboratory of Photocatalysis on Energy and Environment, Fuzhou University, Fuzhou, Fujian. 350002, China; 2 Institute of Advanced Energy Materials, Fuzhou University, Fuzhou, Fujian. 350002, China

## Abstract

An in situ hydrothermal route is developed for fabricating rutile TiO_2_ mesocrystals/reduced graphene oxide nanosheets (TGR) hybrids in the presence of dodecylbenzenesulphonic acid (ADBS). These rutile TiO_2_ mesocrystals with a Wulff shape are composed of ultra-tiny rod-like subunits with the same oriented direction and closely wrapped by the nanosheets of reduced graphene oxide (RGO). It is found that ADBS played a key role for the formation of mesocrystals during the self-assembly process, which pillared the graphene oxide (GO) nanosheets and involved the aggregation of the mesocrystal subunits. Furthermore, the TGR hybrids are used as an anode material and exhibited a large capacity over 150 mA h g^−1^ at 20 C after 1000 cycles, and high rate capability up to 40 C. These high performance characteristics may be due to the intrinsic characteristics of rutile TiO_2_ mesocrystals constructed from ultra-tiny subunits and hybridized with super conductive RGO nanosheets.

Rechargeable lithium-ion batteries (LIBs) are excellent power source for wide range of applications in the portable consumer electronics, such as laptop and mobile phones due to their high-energy storage density, long cycle life and high-power sources^1^,^2^,^3^,^4^,^5^,^6^,^7^,^8^. However, LIBs lack high rate performance and limit their potential applications in the fields of electric vehicle (EV) and hybrid electric vehicle (HEV) because of the use of graphite as an anode material in the present commercial LIBs^7^,^8^,^9^. Thus, it is necessary to develop new anode materials with excellent rate performance, long-term cycling life and safety. In the last decade, much effort has been devoted to developing excellent electrochemical performance, high safe and low-cost metal oxide materials^10^,^11^,^12^,^13^,^14^,^15^,^16^,^17^,^18^. Titanium dioxide (TiO_2_) is one of the most important metal oxides and has been widely applied in many fields. For instance, it is considered as one of the best materials for photocatalysis^19^,^20^ and is also considered as one of the most promising electrode materials for applications in energy storage and conversion^21^,^22^. To date, much attention has been attracted to design various nanostructured TiO_2_ such as nanoparticles^23^, nanosheets^24^,^25^, hierarchical structure^26^,^27^ for LIBs due to their advantages for lithium storage.

Mesocrystals are a new class of superstructured solid materials with a controlled ordered superstructure, often showing single-crystal-like electron diffraction behaviors^28^,^29^,^30^. In brief, we can regard the mesocrystals as an entirely new class of superstructured materials which are constructed of crystallographically oriented nanocrystals. The oriented arrangement leads to an extremely high specific surface area and great many nanoscale pores in mesocrystals, which could provide a space for Li-ion storage on the surface of electrode and a pathway for electrolyte diffusion inside electrode, leading to good electrochemical performances. However, the reports dealing with synthesis of TiO_2_ mesocrystals for LIBs are rare^31^,^32^,^33^. Qi et al^31^ reported the synthesis of spindle-like anatase TiO_2_ mesocrystals composed of nanoparticles along [001] direction; they delivered discharge capacities of 164.9 and 151.7 mA h g^−1^ at 1 and 2 C, which are larger than those of the reported TiO_2_ hollow spheres^34^ and comparable with that of the reported mesoporous spheres^35^. In our previous studies, TiO_2_ mesocrystals constructed from ultrathin rutile TiO_2_ nanowires were synthesized and demonstrated a reversible capacity of 171.3 mA h g^−1^ after 100 cycles at 1 C and retained a capacity of ~100 mA h g^−1^ even at a high current rate of 5 C^32^. However, the intrinsic poor Li-ion and electron conductivity of TiO_2_ limit its rate capability further improved. Therefore, it would be highly desirable to develop TiO_2_ mesocrystals with enhanced conductivity and excellent electrochemical performance. On the other hand, two-dimensional graphene macromolecular sheets of sp^2^ carbon atoms arranged hexagonally have an excellent electrical conductivity^36^ and have also been used to fabricate hybrid graphene-based electrode materials for enhancing properties^37^,^38^,^39^,^40^. Wang et al.^37^ reported the preparation of nanostructured TiO_2_-graphene hybrid materials by using anionic sulfate surfactants in aqueous solutions; these materials showed significantly enhanced special capacities at high charge-discharge rates due to the improved electrode conductivity. Xin et al.^38^ developed a simple route for synthesizing nanostructured TiO_2_/graphene composites which demonstrated superior high-rate charge-discharge capability and cycling stability. The reports about TiO_2_ mesocrystals hybridizing with RGO are limited. Yang et al.^41^ reported that Graphene-TiO_2_ mesocrystal composites exhibited higher photocatalytic activity than pure TiO_2_ mesocrystals and P25. However, there is not reports on fabrication of hybrid electrode materials composed of TiO_2_ mesocrystals-graphene hybrids.

In the present work, hybrid materials of rutile TiO_2_ mesocrystals hybridizing with RGO have been successfully synthesized by an in situ hydrothermal route. The synthesis strategy is schematically depicted in Fig. 1. The well-dispersed GO nanosheets were prepared via oxidized graphite and then dispersed in water by ultra-sonicating. The second step was the formation of an emulsion by dissolved the ADBS in HNO_3_ solution. ADBS played a very important role for preventing stacking of GO nanosheets under strong acidic condition and fabricating the nanostructured rutile TiO_2_ mesocrystals. Furthermore, the hybrids were used as anode materials for LIBs and exhibited large reversible lithium-ion charge-discharge capacity, long-term cycling stability and high-rate capability.

## Results

**Figure 2:**
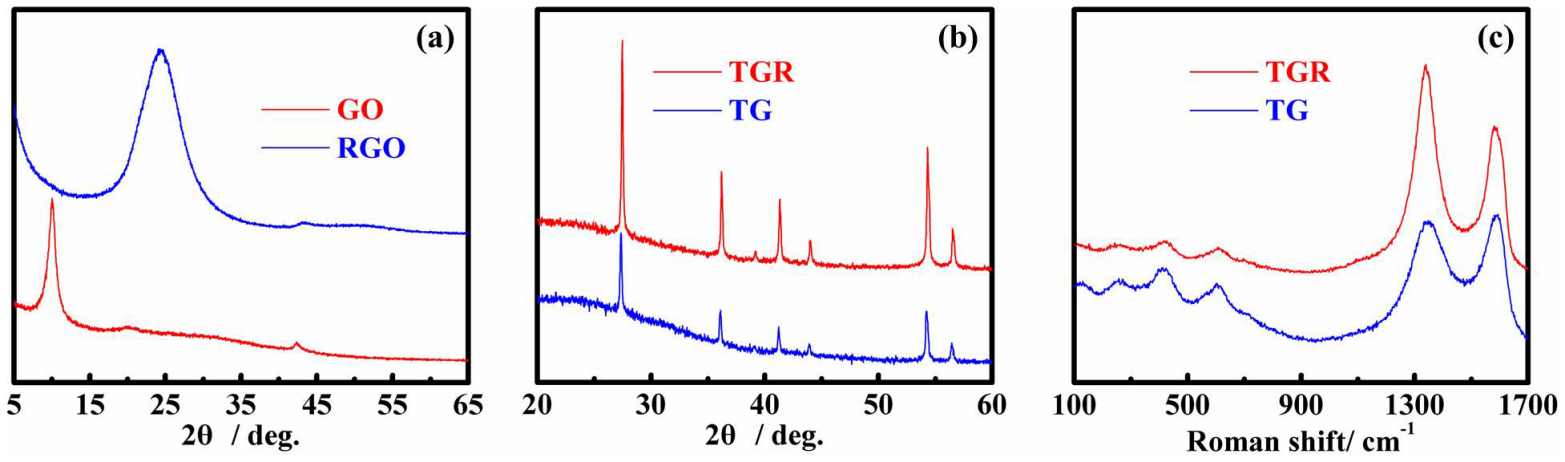
XRD patterns (a) and Raman spectra (b) of TG and TGR hybrids.

Figure 2 shows the XRD patterns of GO and RGO, and the XRD patterns and Raman spectra of TG and TRG hybrids. As depicted in Fig. 2a, a strong diffraction peak of GO can be observed at ~10°, which is can be indexed to the (001) diffraction of GO nanosheets, indicating that the GO nanosheets can be obtained completely feasible by the oxidation of graphite. The peak completely disappears after hydrothermal treatment in water under 140°C for 6 h, accompanying with a new strong and broader peak appearing at ~24.5° (blue) which is due to the (002) diffraction of reduced graphene oxide (RGO). The change of the XRD patterns demonstrate that GO can be fully reduced to RGO via the simple hydrothermal method which will be used in the following reaction. Figure 2b shows the XRD patterns TG and TRG hybrids, which indicates that two samples can be indexed to a tetragonal rutile TiO_2_ (JCPDS 77-0440). It also reveals that the peak intensity of rutile TiO_2_ mesocrystals/reduced graphene oxide nanosheets (TGR) hybrids was stronger than that of rutile TiO_2_ mesocrystals/graphene oxide nanosheets (TG) hybrids, indicating that the crystallinity and size of TiO_2_ mesocrystals were increased obviously after the hydrothermal treatment. GO or RGO was not detected, indicating that they were uniformly dispersed in the final products^40^. Raman spectra of TG and TGR hybrids are shown in Fig. 2c, and the clear identifiable peaks at 250, 441 and 616 cm^−1^ were corresponding to rutile TiO_2_ phase^42^, which were in agreement with the XRD patterns. A couple of peaks at 1360 and 1605 cm^−1^ were assigned to the D-band and G-band of graphene-based materials, respectively. The D-band usually corresponds to a k-point phonon of A_1g_ symmetry, while the G-band is related to the in-plane bond-stretching motion of pairs in sp^2^ carbon atoms. Therefore, the ratio of D/G intensity can be used to estimate the reduction of GO. As can be seen from Fig. 2b, the ratio of D/G intensity was increased significantly, indicating the reduction of GO in the finial sample.

**Figure 3:**
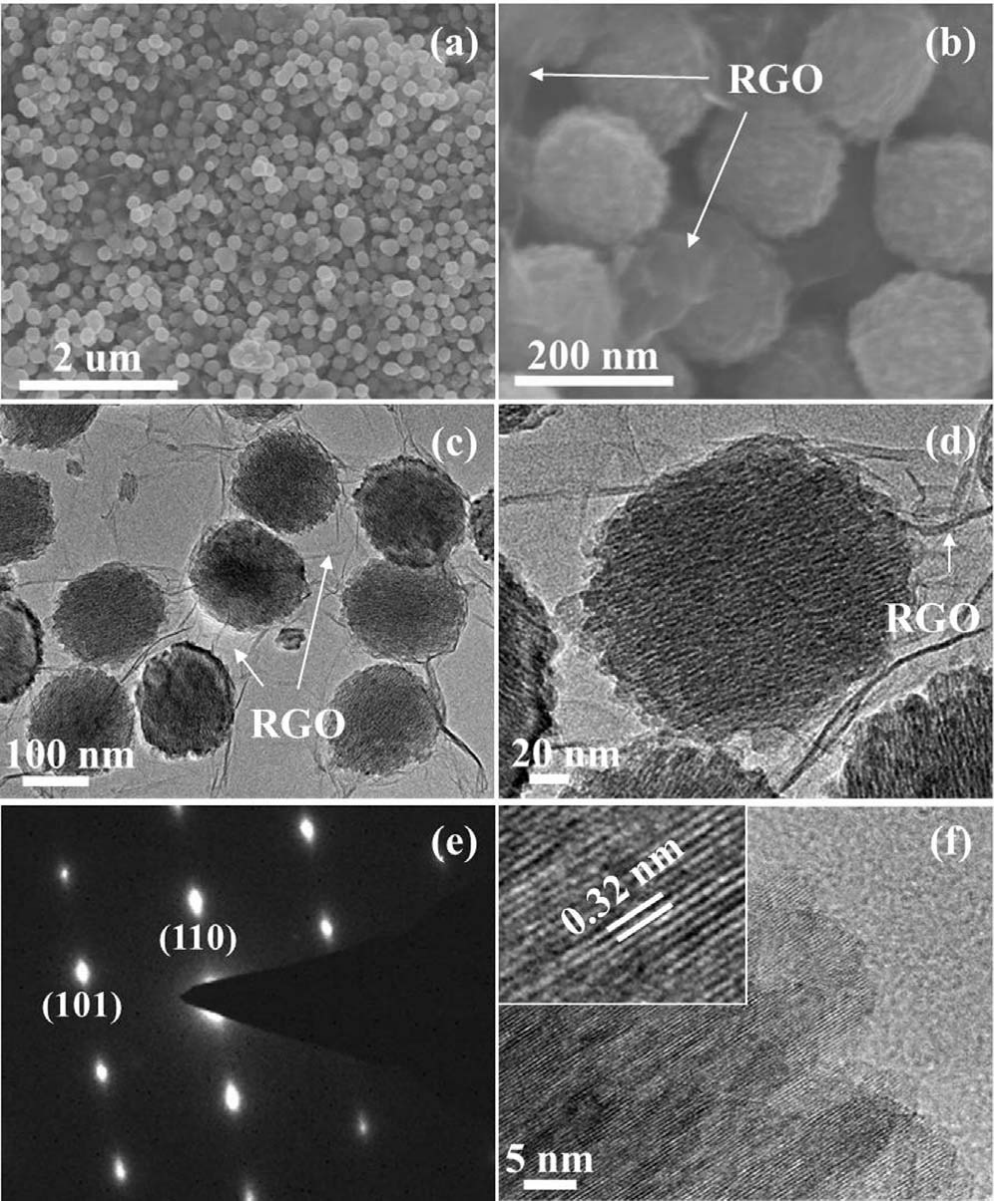
(a, b) SEM, (c, d) TEM and (f) HRTEM images of TGR hybrids, and (e) the SAED pattern taken from a single mesocrystal.

SEM and TEM images of the sample TGR are depicted in Fig. 3. It clear shows that the particles with Wulff shape were quite uniform, and their lengths were 150–200 nm (~180 nm on average), as depicted in Fig. 3a. Figure 3b reveals that the particles have Wulff-shaped nanostructure aggregated by nanoparticles and the sheet-like RGO nanosheets were also observed clear. Figure 3c also shows that the rutile TiO_2_ nanoparticles well dispersed onto RGO nanosheets. On the other hand, it can be found that the Wulff-shaped nanoparticles were wrapped closely by RGO ultrathin nanosheets which were similar to a core-shell structure. At the same time, RGO ultrathin nanosheets bridged nearby nanoparticles together. This is different from the reported data, in which metal oxides were only dispersed on the surface of graphene sheets^43^. A typical high-magnification TEM image in Fig. 3d taken on a single particle reveals that the particle was consisted ultrathin rod-like subunits. It is interesting to find that all of the subunits are oriented along the same direction. At the same time, the internal pores of the particles were clear detected. Lots of elongated pores evenly distributed inside the particles, which caused by the regular arrangement of the rod-like subunits. This result was further confirmed by N_2_ adsorption-desorption measurement, which indicated the presence of rather uniform nanopores with an average size of 4.2 nm (Supplementary Fig. S1). The special surface area and mesoporous volume were 146.2 m^2^ g^−1^ and 0.373 cm^3^ g^−1^, respectively, which larger than the previous reports^31^,^32^. These results suggest that the obtained Wulff-shaped particles were neither a classic monocrystal structure nor a traditional hierarchical structure. The selected area electron diffraction (SAED) pattern (Fig. 3e) exhibits “single-crystal-like” diffraction spots with a slightly elongated shape, suggesting that there was a small lattice mismatch in the assembly in the same orientation, which is a typical structure of mesocrystals^28^,^32^. These results indicate that rutile TiO_2_ mesocrystals were successfully synthesized in the present reaction system. A HRTEM image in Fig. 3f exhibits a well-oriented lattice fringe of 0.32 nm (inset in Fig. 3f), corresponding to d_110_ spacing of rutile TiO_2_ crystal, which in compliance with the analysis of HRTEM image.

**Figure 4:**
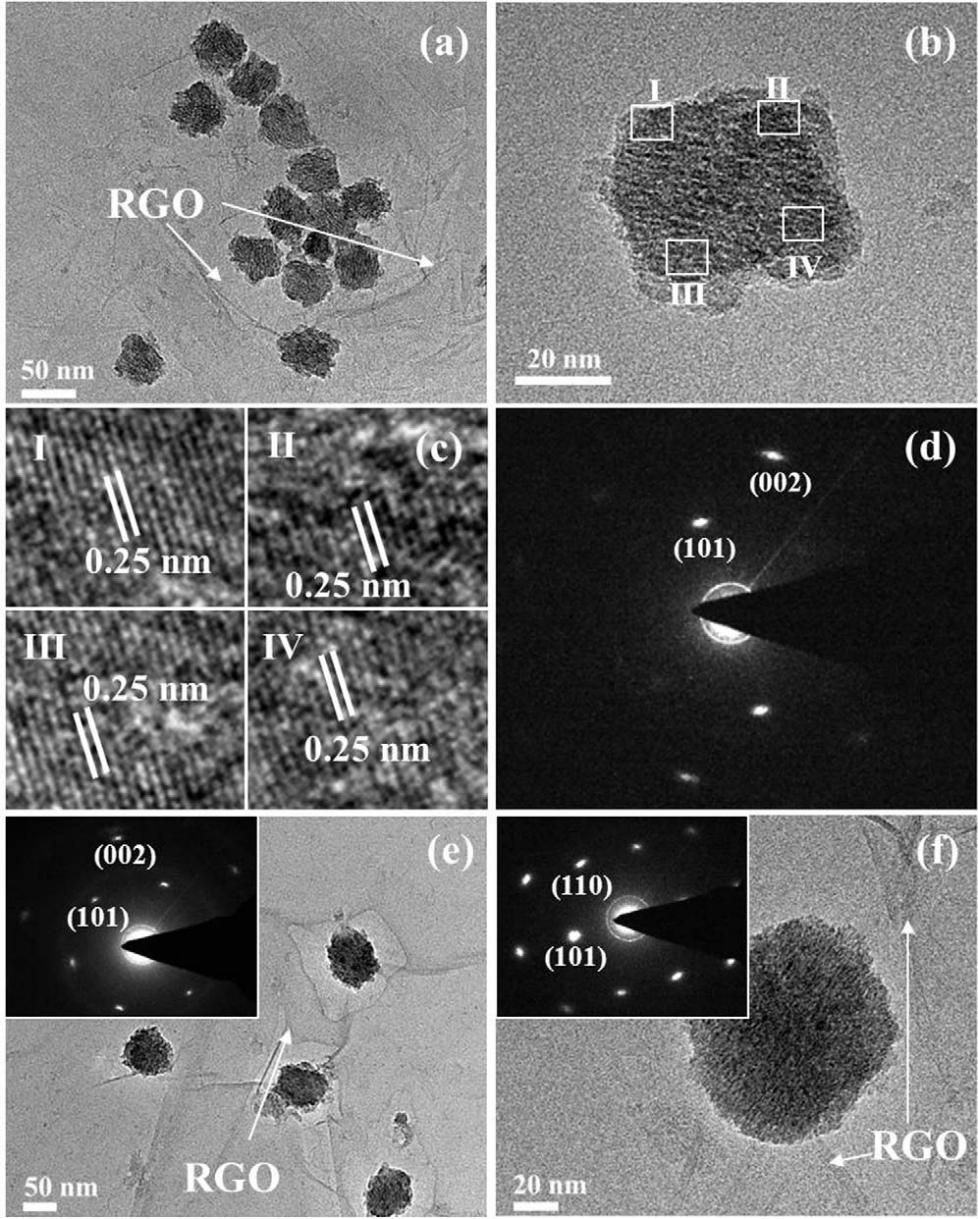
(a–b, e–f) TEM and (c) HRTEM images of TG hybrids obtained at 70°C for different reaction times: (a–d) 6, (e) 12, and (f) 24 h; (d) and insets in Figure e–f are corresponding SAED.

To investigate the formation mechanism of rutile TiO_2_ mesocrystals, a series of experiments were carefully carried out for different reaction times. Figure 4a shows a typical TEM image of TG obtained at 6 h and indicates that TiO_2_ mesocrystals with a uniform size of 30–40 nm were covered by the folding GO nanosheets. A HRTEM image of a single mesocrystal in Fig. 4b confirms that it was composed of ultra-tiny rod-like subunits. HRTEM images (Fig. 4c) taken from four regions (I, II, III, IV in Fig. 4b) indicate that it has a same lattice fringe of *ca.* 0.25 nm in the same orientation direction, corresponding to d_101_ spacing of the rutile TiO_2_. The related SAED image in Fig. 4d reveals a typical diffraction spots of mesocrystals. It is believed that a single-crystal-like mesoscopic structure has been fabricated for a reaction time as short as 6 h. After the reaction time was increased to 12 h, mesocrystals trended to growth and their size was 50–70 nm. It is worthy of mentioning that the obtained samples were little for the reaction time was less than 12 h, indicating the growth of mesocrystal nuclei in this stage. When the reaction time was further increased to 24 h, the shape of mesocrystal was not changed obviously although their size was increased. Meanwhile, it notices that there were not any other tiny particles coexisted with mesocrystals in the selected reaction stages. Thus, the formation of mesocrystals is a self-assembly process based on homoepitaxial mechanism^28^,^44^.

In the present reaction system, ADBS played a key role for fabricating rutile TiO_2_ mesocrystals/RGO hybrids and acted as pillars between the individual graphene oxide nanosheets together with the water molecules which could prevent stacking of GO nanosheets under a strong acidic condition, leaving enough GO nanosheets dispersed to accommodate the TBOT during the hydrolyzing process. More importantly, ADBS was a key factor in fabricating mesocrystals. It is well known that TBOT hydrolyzes easily in the aqueous solution and can be inhibited under a strong acid condition. As mentioned above, HNO_3_ and ADBS were used in the reaction system which could slow down the hydrolysis of TBOT. At the same time, the fast aggregation of Ti-containing colloids could also be prevented. On the other hand, the ADBS connected with Ti-containing colloids could be in favor of lowering the surface energy of the primary nanocrystals and allowed their attachment and assembly into ordered aggregates along a suitable direction. However, the amount of ADBS is not the more the better. A serial reaction based on different amount of ADBS had been carried out carefully, as shown in Supplementary Fig. S2. An inhomogeneous morphology was obtained with a little amount of ADBS, as shown in Supplementary Fig. S2a–b. With the increasing of ADBS, the shape and size of mesocrystal particles became quite uniform. It's worthy to mention that there was scarcely any change of the size even increasing the amount of ADBS to 4 mM. The size of rutile TiO_2_ mesocrystals just increased with the same oriented direction with increasing reaction time under enough ADBS. Porous mesocrystals hybridizing with RGO nanosheets would then be obtained after a hydrothermal treatment at 140°C for 6 h. It is pointed out that mesocrystal structure can be kept after the post-treatments, in which GO was reduced.

**Figure 5:**
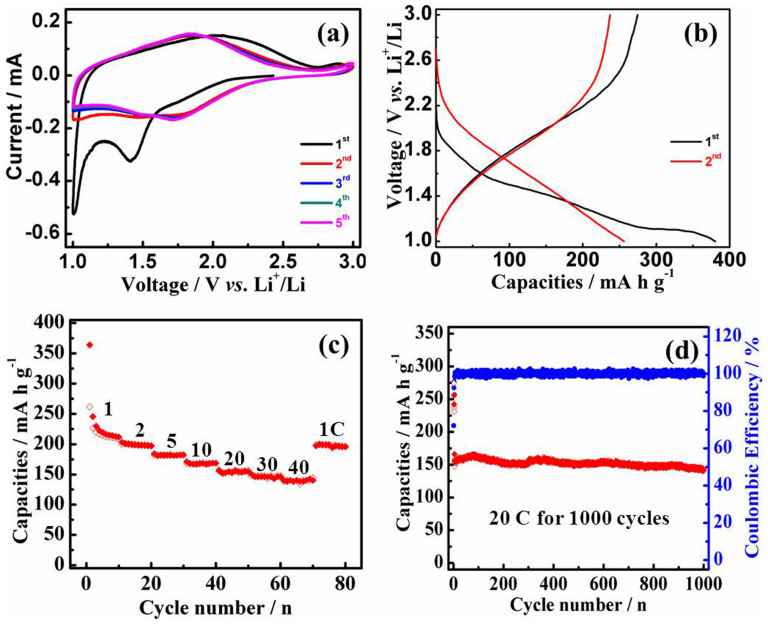
The electrochemical properties of TGR hybrids: (a) cyclic voltammograms between 1.0 and 3.0 V with a scan rate of 0.5 mV s^−1^, (b) charge-discharge profiles at a current rate of 1 C, (c) rate capability from 1 to 40 C, (d) cycling performance at a current rate of 20 C after activating at 1 C for 3 cycles.

Recently, there has been a great deal of interest in using nanostructured mesocrystals as electrode materials in LIBs, especially TiO_2_ mesocrystals. However, the poor lithium ions and electrons conductivity of TiO_2_ limits its rate capability improved. In the present work, rutile TiO_2_ mesocrystals hybridized with RGO nanosheets were fabricated in order to enhance the conductivity of lithium ions and electrons. As shown in Fig. 5a, the representative CV curves of TGR hybrids were different slightly from the previously reported data^32^. A general cathodic peak at appropriately 1.45 V and a very sharp cathodic peak approaching 1.0 V in the first cycle were attributed to the lithium-ion insertion into the rutile structure, while its associated anodic reaction would be identified as a broad peak at about 2.0 V. In the subsequent cycles, a pair of broad cathodic and anodic peak at 1.55 and 1.75 V were observed, showing a well reversible electrochemical reaction. At the same time, we examine the CV at different scan rates, as shown in Supplementary Fig. S3. Broad CV peaks between 1.5 and 2.5 V are observed, indicating that lithium-ion intercalation into and deintercalation out of the TGR may be a pseudocapacitive process. Figure 5b shows the charge-discharge profiles of TGR hybrids at a current rate of 1 C based on the theoretical capacity of rutile TiO_2_ (168 mA g^−1^) for the initial two cycles between 3.0 to 1.0 V. A large capacity of 380.4 mA h g^−1^ was achieved at the first discharge cycle with a corresponding charge capacity of 274.5 mA h g^−1^ based on the weight of rutile TiO_2_ mesocrystals. It can be clear observed that there was a voltage plateau at near 1.1 V in the first discharge profile, which was in agreement with the analysis of the first cycle in CV measurement. According to a similar result reported in the literature^45^,^46^, this can be attributed to the irreversible change in the structure of the rutile TiO_2_ upon deeper lithium-ion insertion. However, it delivered a Coulombic efficiency as high as 72%, which was much higher than other graphene-based^39^ or carbon-coated^47^ rutile TiO_2_ electrode materials. The rate capability of TGR hybrids was evaluated by charge-discharge at various current rates from 1 to 40 C (Fig. 5c and Supplementary Fig. S4). It clear reveals that the cell made of the TGR hybrids shows excellent cycling capacity retentions at each current rate. A reversible capacity of 215.0 mA h g^−1^ was obtained after 10 cycles at 1 C. Remarkably, a large reversible capacity of 139.6 mA h g^−1^ can be achieved even at a current rate as high as 40 C. Importantly, the capacity can be returned to the initial value at 1 C after measured at high current rates, indicating that TGR hybrids delivered a high-rate performance. Furthermore, it is surprising to find that the TGR hybrids demonstrated an excellent long-term cycling stability at a current rate as high as 20 C after activating at 1 C for initial three cycles. As shown in Fig. 5d, an average capacity of 155.7 mA h g^−1^ was achieved for the initial 500 cycles and could be still retained 152.2 mA h g^−1^ after 1000 cycles (only ~0.01% per cycle from cycle 4^th^ to 1000^th^ cycle). It also exhibited a high Coulombic efficiency with an average value of almost 100.0% over 1000 cycles. For estimating the increased capacity of RGO nanosheets, pure RGO nanosheets were used as an anode material and its electrochemical performance was measured at a current rate of 20 C with a same voltage window of 1.0–3.0 V. An average capacity was found to be *ca.* 30 mA h g^−1^ for 300 cycles (Supplementary Fig. S5). As described in Supplementary Fig. S6, RGO content in TGR hybrids was only 9.7%, the capacity contribution from RGO was estimated to be *ca.* 3 mA h g^−1^ with respect to TiO_2_ which could be ignored.

**Figure 6:**
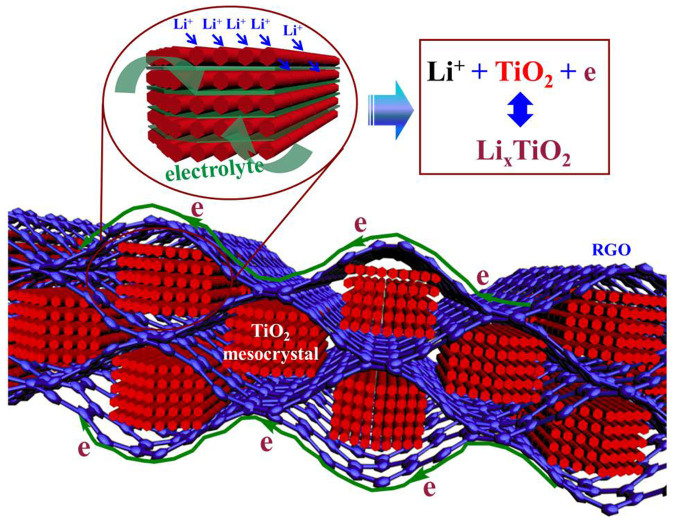
Schematic illustration of transport pathway of lithium ions and electrons in the channel of the TGR hybrids.

Therefore, the cell made of TGR hybrids exhibited large capacity, remarkable rate capability and outstanding cycle stability may be caused by the synergistic coupling effects between the rutile TiO_2_ mesocrystals and RGO nanosheets, as presented in Fig. 6 and described the following: (i) rutile TiO_2_ mesocrystals composed of ultra-tiny nanoparticle subunits drastically shorten the transport distance of lithium ions which greatly facilitate lithium-ion intercalation into rutile TiO_2_; (ii) rutile TiO_2_ mesocrystals with a porous structure can permit facile diffusion of the electrolyte and also promote Li-ions and electrons diffusion through an electrode film; (iii) the unique mesoscopic structure with pores hybridizing with ultra-thin RGO nanosheets provides a large surface area, leading to a high contact area with the electrolyte and also improves the storage ability of lithium ion; (iv) the ultra-thin reduced graphene oxide nanosheets can act as a super conductive substrate for enhancing the electron transport. In a word, the shorten diffusion distance of Li-ion by unique mesocrystalline structures and enhanced conductive ability of electrons through recombination by RGO are extremely beneficial for energy storage, leading to large capacity, excellent cycling stability and high rate capability.

## Discussion

An *in situ* hydrothermal route is developed for fabricating hybrids of TGR. It is demonstrated that the ADBS additive played a key role during the self-assembly process, which pillared the GO nanosheets and involved the aggregation of the mesocrystal subunits. Furthermore, hybrids of TGR are used as an anode material for Li-ion intercalation reaction and exhibit a large capacity, excellent cycling stability and high rate performance. These high performance characteristics may be due to the intrinsic characteristics of rutile TiO_2_ mesocrystals constructed from ultra-tiny subunits and hybridized with super conductive reduced graphene oxides nanosheets, in which the porous structure can permit facile diffusion of the electrolyte. They can also enhance the contact between the electrode surface and the electrolyte, while the ultra-thin subunits can shorten the transport distance of Li-ions and electrons during electrochemical cycling. On the other hand, the conductive ability of the cell made of TGR hybrids is significantly improved. At the same, the porous mesocrystal can also accommodate volume changes in the charge-discharge process. Therefore, we believe that the present synthetic route can be further extended to produce other mesocrystals/graphene hybrid materials with promising applications in photocatalysis, supercapacitor and dye-sensitized solar cell.

## Methods

### Synthesis of GO

Graphene oxide (GO) nanosheets were prepared starting from natural graphite powder (325 mesh) by using a modified Hummers method which is the most commonly used approach currently^48^. Briefly, 4.0 g of natural graphite powder was added slowly into 100 mL of a mixed concentrated H_2_SO_4_/H_3_PO_4_ with a volume ratio of 9:1 (90:10 mL) under vigorous stirring in an ice bath. Ten minutes later, 15.0 g of KMnO_4_ was added pinch by pinch into the black liquid with keeping the temperature below 20°C. Subsequently, the reaction was then heated to 35–40°C and kept with vigorous stirring for 3 h. After that, 750 mL of deionized water was poured into the mixture and then 40 mL of 30% H_2_O_2_ were added, too. The mixture was then washed in succession with 5% HCl (2 L), and deionized water till the pH value increasing to 6–7 and then dialyzed for a week. The final material was dried overnight at 70°C. For the easily using in the subsequent reaction, the GO nanosheets was dispersed into distilled water (5 mg mL^−1^) followed ultra-sonicating sufficiently.

### In situ Synthesis of Rutile TiO_2_ Mesocrystals/GO

Hybrid material of rutile TiO_2_ mesocrystals/GO (denoted as TG) was synthesized by an *in situ* hydrothermal route. Typically, 2 mM of ADBS was dispersed in 70 mL of 2 M HNO_3_ solution. After stirring for 30 min, 10 mg of GO was dropwise into the milk-white suspension with vigorous stirring for 60 min. Finally, 3 mM of tetrabutyl titanate (TBOT) was dropwise into the suspension and then kept stirring at 70°C for 2 days. After cooled to room temperature, the product was washed extensively with deionized water and ethanol for several times and then dried at 70°C for 6 h in air. For reduce the GO nanosheets, 100 mg of TG sample was dispersed in 35 mL of H_2_O. After stirring for 30 min, the resulting solution was transferred into a Teflon-lined stainless steel autoclave with a capacity of 50 mL, and then was kept at 140°C for 6 h. After cooling to room temperature, TGR hybrids was washed with deionized water and ethanol for several times and then dried at 70°C 6 h in air.

### Characterizations

X-ray diffraction (XRD) patterns were recorded on a PANalytical X'Pert spectrometer using the Co Kα radiation (λ = 1.78897 Å), and the data were changed to Cu Kα data. Raman spectroscopic measurements were performed on a Renishaw inVia Raman System 1000 with a 532 nm Nd:YAG excitation source at room temperature. Scanning electron microscopy (SEM, S4800 instrument) and Transmission electron microscopy (TEM, FEI F20 S-TWIN instrument) were applied for the structural characterization of the samples. N_2_ adsorption-desorption analysis was measured on a Micro-meritics ASAP 2020 instrument (Micromeritics, Norcross, GA, USA). The specific surface area, pore size distributions and specific volume of the as-prepared samples were analyzed using the Barrett Joyner Halenda (BJH). To determine the actual amount of GO or RGO in the hybrids, thermogravimetric analysis (TGA) was performed using a CHNS/O analyzer (PE 2400II, Perkin Elmer, America) in air atmosphere.

### Electrochemical measurements

For the electrochemical measurement of lithium-ion intercalation, the TGR hybrids were admixed with polyvinylidene fluoride (PVDF) binder and acetylene black carbon additive in a weight ratio of 80:10:10, respectively. The mixture was spread and pressed on copper foil circular flakes as working electrodes (WE), and dried at 110°C in vacuum overnight. The mass of the active material is 0.5 mg cm^−2^. Lithium foils were used as the counter electrodes. The electrolyte was 1 M LiPF_6_ in mixture of ethylene carbonate (EC), ethylene methyl carbonate (EMC) and dimethyl carbonate (DMC) with a volume ratio of 1:1:1. The separator was Celgard2400 (America) micro-porous polypropylene membrane. The cells were assembled in a glove box filled with highly pure argon gas (O_2_ and H_2_O levels < 1 ppm), and charge-discharge tests were performed in the voltage range of 1.0 to 3.0 V (*vs.* Li^+^/Li) at different current rate on a Land automatic batteries tester (Land CT 2001A, Wuhan, China).

## Author Contributions

T.B.L. and M.D.W. proposed and designed the experiments. T.B.L. and H.Y.Q. carried out the synthetic experiments and conducted the characterization. T.B.L., J.Y. and F.Y.X. performed the HRTEM, SEM characterization and structural analysis. T.B.L. and M.D.W. analysed the data. T.B.L. and M.D.W. wrote the manuscript. All the authors participated in discussions of the research.

## Supplementary Material

Supplementary Information Rutile TiO_2_ Mesocrystals/Reduced Graphene Oxide with High-Rate and Long-Term Performance for Lithium-Ion Batteries

## Figures and Tables

**Figure 1:**
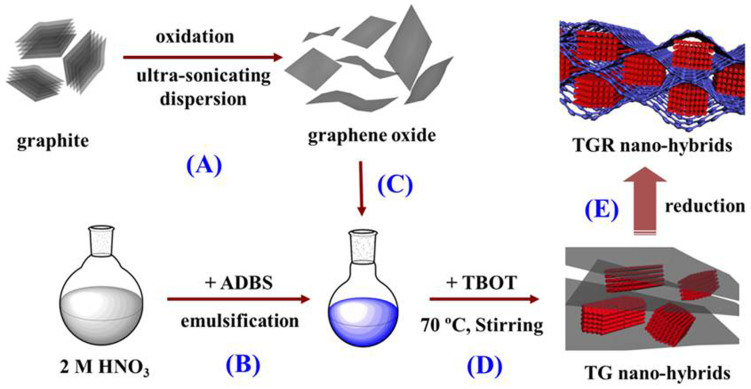
Schematic illustration for the formation of TGR hybrids.
