# Supplementary material for: Rutile TiO2 Mesocrystals/Reduced Graphene Oxide with High-Rate and Long-Term Performance for Lithium-Ion Batteries
Source: Sci Rep. 2015 Feb 17;5:8498. doi: 10.1038/srep08498 (PMC4330539; doi:10.1038/srep08498)
Supplement: Supplementary Information — Rutile TiO2 Mesocrystals/Reduced Graphene Oxide with High-Rate and Long-Term Performance for Lithium-Ion Batteries [file srep08498-s1.doc]

**Supplementary Information**

**Rutile TiO_2_ Mesocrystals/Reduced Graphene Oxide with High-Rate and Long-Term Performance for Lithium-Ion Batteries**

Tongbin Lan, Heyuan Qiu, Fengyan Xie, Jie Yang, Mingdeng Wei *

* State Key Laboratory of Photocatalysis on Energy and Environment, Fuzhou University, Fuzhou, Fujian 350002, China; Institute of Advanced Energy Materials, Fuzhou University, Fuzhou, Fujian 350002, China; E-mail: wei-mingdeng@fzu.edu.cn

**Supplementary Figure S1│** N_2_ adsorption–desorption isotherms of the TGR nano-hybrids; the inset is the BJH pore size distributions from the adsorption branch.

**Supplementary Figure S2│**SEM images of TG hybrids obtained at a temperature of 70 °C with different amount of ADBS: (a-b) 1, and (c-d) 4 mM.

**Supplementary Figure S3│**CV curves at different scan rates of TGR hybrids.

**Supplementary Figure S4│**Charge-discharge profiles at different current rates from 1 to 40 C.

**Supplementary Figure S5│** Cycling performance of RGO nanosheets at a current rate of 20 C after activating at 1 C for 3 cycles. The voltage is between 1.0 and 3.0 V.

**Supplementary Figure S6│** TGA curves of TG (blue) and TGR (red) nano-hybrids, respectively.
